# Authorship attribution based on Life-Like Network Automata

**DOI:** 10.1371/journal.pone.0193703

**Published:** 2018-03-22

**Authors:** Jeaneth Machicao, Edilson A. Corrêa, Gisele H. B. Miranda, Diego R. Amancio, Odemir M. Bruno

**Affiliations:** 1 Scientific Computing Group, São Carlos Institute of Physics, University of São Paulo, PO Box 369, 13560-970, São Carlos, São Paulo, Brazil; 2 Institute of Mathematics and Computer Science, University of São Paulo, Avenida Trabalhador são-carlense, 400, 13566-590, São Carlos, São Paulo, Brazil; University of Rijeka, CROATIA

## Abstract

The authorship attribution is a problem of considerable practical and technical interest. Several methods have been designed to infer the authorship of disputed documents in multiple contexts. While traditional statistical methods based solely on word counts and related measurements have provided a simple, yet effective solution in particular cases; they are prone to manipulation. Recently, texts have been successfully modeled as networks, where words are represented by nodes linked according to textual similarity measurements. Such models are useful to identify informative topological patterns for the authorship recognition task. However, there is no consensus on which measurements should be used. Thus, we proposed a novel method to characterize text networks, by considering both topological and dynamical aspects of networks. Using concepts and methods from cellular automata theory, we devised a strategy to grasp informative spatio-temporal patterns from this model. Our experiments revealed an outperformance over structural analysis relying only on topological measurements, such as clustering coefficient, betweenness and shortest paths. The optimized results obtained here pave the way for a better characterization of textual networks.

## Introduction

The current massive production of data has brought up plenty of challenges to the areas of Data Mining, Natural Language Processing (NLP) and Machine Learning. An example of a current challenge in information sciences is the authorship attribution task, which amounts to the ability to assign authorship to anonymous or disputed documents. This task has drawn attention from researchers mostly for its implications in real applications, such as plagiarism detection [1, 2], forensics against cyber crimes [3] and resolution of disputed documents [4].

Several methods have been proposed to undertake the authorship attribution problem [4]. Traditional techniques use text analytics and natural language processing concepts to characterize authors’ writing styles [4]. For example, in several studies, it has been shown that the raw frequency of function words or the intermittency of content words is notably useful to discriminate authors’ styles [5, 6]. In recent years, deeper paradigms have been employed to tackle this problem. Syntactical and semantical features are some examples of features not relying only on simple statistical analyses [7]. Despite being effective in particular contexts, deeper paradigms require a more complex data handling, a painstaking effort that may not yield good results in generic scenarios. Even though methods based on simple statistical analyses yield, in general, excellent results with the advantage of not requiring a large corpora for training or language-dependent resources, they are prone to manipulation via obfuscation or imitation attacks [6, 8]. For this reason, more robust statistical methods have been proposed.

A recent trend in authorship attribution research is using the complex network framework, due to the success of its use in related tasks, mostly in text classification tasks [9–15]. In this paradigm, documents are modeled by means of a co-occurrence network [12], and the properties of the formed networks are used as authors’ fingerprints in the classification process [16]. Although such methods have proven useful for discriminating writing styles with a certain robustness provided by topological analysis, they usually provide no better results than traditional techniques based e.g. in n-grams models when used as a single source of text characterization. Complex network topologies, on the other hand, are less prone to direct manipulation, since the attacker cannot easily manipulate complex network features, which are mostly dependent on the interaction among all words in the text. Note that complex network-based measurements provide a complementary view of unstructured documents, a feature that can be further explored in hybrid approaches.

In a typical networked-based authorship recognition system, texts are modeled as a network and the structure of these networks is then used as a relevant feature to discriminate distinct authors [16]. While structural measurements are useful to understand the main topological properties of texts, they may provide an ambiguous characterization, mainly when subtleties in style are not mapped into equivalent informative network structures. For this reason, the creation of informative, efficient and unambiguous network measurements for specific models remains as an open problem in network science. In this context, we explore a novel network characterization based on cellular automata theory (CA) [17].

In the last decade, network science and cellular automata were combined in several applications [18–21]. This discrete dynamical system, called as network automata, uses the network structure as the tessellation of the cellular automaton, whose dynamics is governed by a rule that defines the states of its nodes at each time step. Network automata turned out to be a powerful tool for pattern recognition purposes because it combines the advantages of the networks for modeling with the capabilities of CAs to extract complex patterns [21, 22].

In this manuscript, we propose a method to characterize networks representing written texts to tackle the authorship attribution task. The proposed method is based on Life-Like Network Automata (LLNA) [21], which was inspired by the 2D Life-like CA [23], a well-known set of rules explored in diverse fields [24–27]. We depart from the well-known word-adjacency model and include a LLNA dynamics to characterize text networks. More specifically, our approach relies on a selection of informative LLNA’s rules and, therefore, we expect to obtain spatio-temporal patterns possessing two important properties: (i) the books written by the same author displays similar patterns; and (ii) books written by distinct authors display distinct spatio-temporal patterns. Using a collection of texts written by 8 authors, we obtained an accuracy of 70.5%, which is considerably more accurate than structural methods based solely on topological properties of networks and, therefore, demonstrating the good performance of the proposed method.

## Related work

The first methods designed to automatically identify the authorship of disputed documents were proposed in the 19th century, with Mendenhall’s work applied to analyze Shakespeare’s plays [28]. Dominated by statistical and linguistic analysis, this area has since evolved into what we now define as authorship attribution task. Differently from what was done in early days, currently, the analysis has been mostly carried out through computational methods rather than using human experts [4].

Most methods propose the characterization of an author through the quantification of specific features, trying to capture the authors’ writing style [4]. Some simple features are: vocabulary size, frequency of function words and characters and the length of words and sentences. More complex features that require the use of advanced natural language processing techniques are: semantic features (e.g. semantic dependencies) [29], syntactic features (e.g. part-of-speech, sentence and phrase structure) [30] and other features relying on specific linguistic concepts [7]. All these features can be used as input to machine learning methods [4] or linguistic profiling techniques [7] to classify and identify authors.

Following a current trend in NLP and other areas of science [31], the authorship attribution task has been tackled using the complex network framework [5, 12, 15, 16, 32–35]. This approach usually consists of modeling entities of a problem as vertices and the interaction between these entities as edges. After the modeling phase, the final structure generated by this process can be characterized by several topological/dynamical measurements [36], which in turn can be used to feed a machine learning method [37]. The co-occurrence network [12] is a clear example of modeling texts into complex networks that has been explored in several works [5, 16, 38, 39] and even refined in some cases, such as networks based only on function words [32]. Although there are alternative models such as syntactic dependency and semantic networks, most of the recent works have kept the use of co-occurrence modeling given its simplicity and little dependence on linguistic resources (a deep limitation in some languages).

All of the network-based models mentioned above have demonstrated their ability to perform the authorship attribution task. Even though such methods can not be compared directly, given the use of different datasets, such methods still perform poorly when compared to traditional statistical methods. However, as shown in some studies [32, 39], the characteristics captured by these models are different from the traditional ones. Most importantly, when traditional statistical methods are combined with those based on network features, improved results can be obtained. This fact justifies the creation of more sophisticated techniques for the characterization of networks. Following this idea, in this work, instead of using structural measures commonly used in complex networks, we chose to characterize networks through methods based on cellular automata.

## Material and methods

### Proposal overview

**Fig 1:**
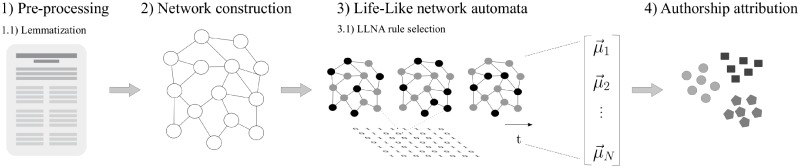
Authorship attribution framework based on LLNA method. The following steps are applied: (1) a written text is pre-processed; (2) a network is generated based on the extraction of keywords from the pre-processing; (3) a selected LLNA rule evolves over the textual network topology; (4) spatio-temporal features from the LLNA are extracted and then are used for the authorship attribution task.

In this section, we introduce an overview of the main proposal (see Fig 1) to understand not only the sequence of mathematical preliminaries, but also the experiments setup that are presented in Results and discussion section. First, we introduce the well-known network model of text representation, the word-adjacency model. We also present optional text pre-processing strategies, which may be applied to improve the characterization of texts. Some network measurements used to explore the properties of networks are presented. Next, we discuss the Life-Like network automata representation used in this article and their respective measurements. The measurements extracted from the Life-Like network automata dynamics are then used to characterize the style of each author.

### Modeling and characterizing texts as networks

In recent years, distinct ways to model texts as complex networks and graphs have been proposed [40]. Particularly, in the current study, we have used the so-called word adjacency (or co-occurrence) model, as it has been proven useful to grasp stylistic textual patterns [16, 41, 42]. In this model, each node represents a word and the edges are created whenever two words appear as adjacent in the text. Note that even the last word of a sentence can be linked to the first word of the next sentence, since punctuation marks are removed from the analysis (see next section). Mathematically, the word adjacency network is represented by an adjacency matrix *A*, whose elements *A*_*ij*_ are defined as

$$\begin{array}{c} A_{ij}=\{\begin{array}{cc} 1, & \text{if}\;i\;\text{and}\;j\;\text{are}\;\text{connected},\text{and}\\ 0, & \text{otherwise}. \end{array} \end{array}$$ (1)

#### Network construction

**Fig 2:**
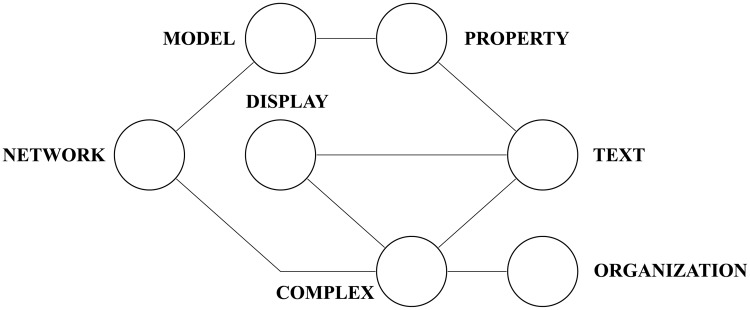
Exemplification of the network modeling. Here was used a short text *“Complex networks model several properties of texts*. *A complex text displays a complex organization”*. In this example, we considered the lemmatization of all words to construct the network.

Prior to the transformation of the text as a network, some pre-processing steps may be required. In most of the applications devoted to represent texts as networks, the three following steps are performed. The first step is the tokenization, which is responsible to split the document into meaningful units, such as words and punctuation marks. The second step performs the removal of stopwords, which are the words conveying little semantic meaning such as articles and prepositions. The list of stopwords is shown in S1 File of the Supplementary Information. Note that, in this phase, punctuation marks are also disregarded, as they do not contribute to the semantic meaning of text. Finally, the third step, a lemmatization is applied to map the remaining words into their canonical forms. As such, verbs and nouns are mapped to their infinitive and singular forms, respectively. The lemmatization process usually requires the identification of the individual parts-of-speech to solve possible ambiguities. In this paper, we have used the Average Perceptron part-of-speech Tagger proposed by Collins [43]. An example of the pre-processing steps applied to a given short text *“Complex networks model several properties of texts*. *A complex text displays a complex organization”* and its corresponding network construction is illustrated in Fig 2 and S2 File of the Supplementary Information.

Although lemmatization is often used in NLP tasks, Toman [44] argued that this pre-processing step does not affect the performance of general text classification systems. To our knowledge, there is no systematic analysis on the effect of lemmatization on network-based authorship recognition methods. For this reason, we have considered the following three variations in the application of pre-processing in raw texts: (i) *none*, no lemmatization is performed; (ii) *partial*, only nouns are lemmatized; and (iii) *full*, all words are lemmatized, as it is done in more traditional works. We have considered full lemmatization in (iii) over stemming because the lemmatization is a more informed technique to normalize words. Differently from stemming methods, the lemmatization can solve ambiguities in the normalization process by using the part-of-speech of the words.

#### Network measurements

In this section, we present a brief description of measurements used to characterize the topological properties of complex networks. These measurements are used here to study how the properties of text networks vary with distinct pre-processing steps. In addition, these measurements are also used for comparison and validation purposes.

The simplest measurements are the number of nodes (*N*) and edges (*E*). The density of a network is defined as *d* = *E*/*N*(*N* − 1), i.e. the fraction between the total number of edges and the maximum possible number of edges obtained in an equivalent fully connected network.

The degree *k*_*i*_ of a node *i* is defined as the number of neighbors that *i* and is given by

$$\begin{array}{c} k_{i}=\sum_{j=1}^{N}A_{ij}. \end{array}$$ (2)

The coefficient *γ* of the degree distribution *P*(*k*) = *k*^−*γ*^ is another widely known measurement in network science [45]. Similar to other real-world networks, text adjacency networks display the scale-free behavior [12]. To estimate the coefficient *γ*, we used the strategy defined in [46]. The degree is also usually measured in global terms as

$$\begin{array}{c} \left\langle k\right\rangle =\frac{1}{N}\sum_{i=1}^{N}\sum_{j=1}^{N}A_{ij}. \end{array}$$ (3)

The quantity defined in Eq 3 is the average degree, a measurement that has been applied in a myriad of network contexts [45], even though many of the studied distributions makes this quantity not a representative element of the distribution, as many networks display a fat-tailed degree distribution [47–50]. This is the case of text networks, whose fat-tailed degree distribution stems from the Zipf’s law [51]. However, in several cases, the average degree is useful to discriminate distinct topologies [45].

Another well known connectivity measurement is the hierarchical degree *k*^*h*^, which corresponds to the number of neighbors *h* nodes away from the reference node. This is a simple extension of the concept of node degree for further hierarchies. Despite its seeming simplicity, the use of hierarchies has proven useful to improve the characterization of several real-world networks.

While the degree is essentially a local measurement, some other indexes were specially devised to characterize the global topology of networks. This is the case of distance-based metrics. Measurements based on geodesic paths include the average shortest path length (〈*L*〉) and the diameter (*D*). The average shortest path length of a network is computed as

$$\begin{array}{c} \left\langle L\right\rangle =\sum_{i=1}^{N}\sum_{j=1}^{N}\frac{d_{ij}}{N(N-1)}, \end{array}$$ (4)

where *d*_*ij*_ is the length of the shortest distance between nodes *i* and *j*. The diameter of a network *D*, is the largest path length among all distances.

The transitivity of the network was measured by the average clustering coefficient 〈*C*〉 = 1/*N* ∑ *cc*_*i*_, where *cc*_*i*_ is the clustering coefficient computed for node *i*. This quantity measures the probability of any two neighbors of *i* being linked. Mathematically, the local clustering coefficient is computed as

$$\begin{array}{c} cc_{i}=\frac{2e_{i}}{k_{i}\left(k_{i}-1\right)}, \end{array}$$ (5)

where *e*_*i*_ represents the number of edges between the neighbors of node *i*. Even though this measure was originally used in social sciences, the clustering coefficient has been used to identify the specificity of words in distinct contexts.

Finally, we used the assortativity measure to measure if similar nodes are connected to each other. In this case, we used the concept of degree correlation, which assigns a high assortativity value for networks with edges established mostly between nodes with similar degree [52]. The assortativity is given by

$$\begin{array}{c} \Gamma =\frac{\left(1/E\right)\sum_{j>i}k_{i}k_{j}A_{ij}-{\left[\left(1/E\right)\sum_{j>i}\left(1/2\right)\left(k_{i}+k_{j}\right)A_{ij}\right]}^{2}}{\left(1/E\right)\sum_{j>i}\left(1/2\right)\left(k_{i}^{2}+k_{j}^{2}\right)A_{ij}-{\left[\left(1/E\right)\sum_{j>i}\left(1/2\right)\left(k_{i}+k_{j}\right)A_{ij}\right]}^{2}}\;. \end{array}$$ (6)

In general, text networks are disassortative, i.e. Γ < 0 [12].

### Life-Like Network Automata

A network automata can be defined as a tuple $C=\langle T,S,s,s_{0},\phi \rangle$. T represents the network automata space, which is the topology of a network comprising *N* nodes (cells). S is the set of binary states *s*_*i*_, where *s*_*i*_ = 1 is the live state and *s*_*i*_ = 0 the dead state. The cell’s state can be identified by the function *s*, such that *s*(*c*_*i*_, *t*) gives the state of cell *c*_*i*_ at time *t*. Finally, *s*_0_ represents the initial configuration of all cells (at *t* = 0) and *ϕ* is a transition function, i.e., the rule that governs the network automata dynamics by defining how the states of the cells are updated over time [21].

The LLNA, a powerful tool for pattern recognition [21], was proposed as a class of binary network automata inspired by the rules of the Life-like Cellular Automata (CA), which uses a set of outer-totalistic rules, *i.e.,* rules that depend on the current state of cell *c*_*i*_ and on the states of neighboring cells (i.e., the neighborhood density). The LLNA transition function is stated as

$$\begin{array}{c} s\left(c_{i},t+1\right)=\{\begin{array}{cc} 1, & \text{if}\;s\left(c_{i},t\right)=0\;\text{and}\;x/r\leq \rho_{i}<\left(x+1\right)/r\Rightarrow \text{born}\;\left(B\right)\;\text{rule}\\ 1, & \text{if}\;s\left(c_{i},t\right)=1\;\text{and}\;y/r\leq \rho_{i}<\left(y+1\right)/r\Rightarrow \text{survive}\;\left(S\right)\;\text{rule}\\ 0, & \text{otherwise}, \end{array} \end{array}$$ (7)

where the neighborhood density *ρ*_*i*_ of node *i* is the proportion of alive neighbors, i.e.

$$\begin{array}{c} \rho_{i}=\frac{1}{k_{i}}\sum_{j=1}^{N}A_{ij}s\left(c_{j},t\right) \end{array}$$ (8)

and *k*_*i*_ the degree defined in Eq (2).

In Eq (7), variables *x* and *y* represent the conditions of the Life-Like rule which are stated in the form B*x*_0_ *x*_1_ … *x*_8_-S*y*_0_ *y*_1_ … *y*_8_, where B and S stand for “birth” and “survival”, respectively; and *x*_*i*_ and *y*_*i*_ varies from 0 to 9, for instance B3-S23 (see S3 File of the Supplementary Information). Moreover, given that the LLNA is based on Moore’s neighborhood, defined by a central cell and its eight nearest neighbors, the constant *r* = 9 accounts for all the combinations of the Life-Like family. Therefore, there exists a total of 2^9+9^ = 262, 144 possible transition rules, which provides a vast space of optimal solutions for a specific problem [21].

### LLNA spatio-temporal pattern

**Fig 3:**
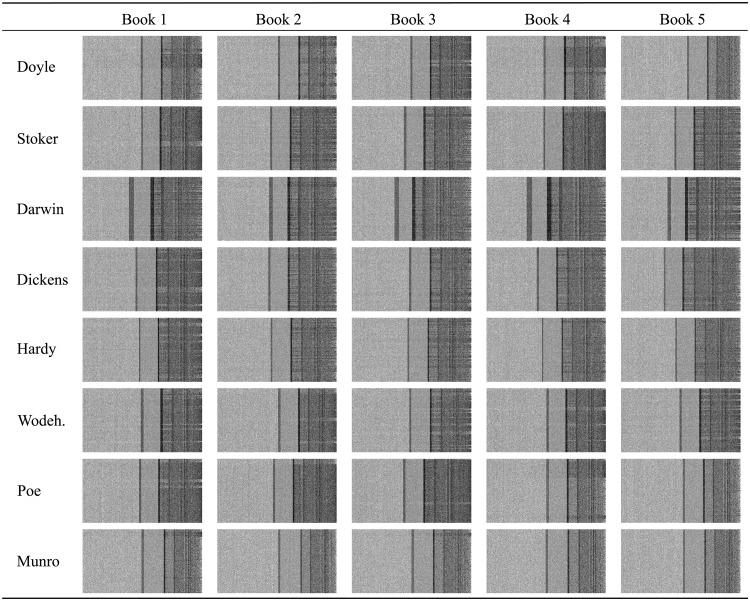
Spatio-temporal diagrams. Here was used the LLNA rule B2478-S25 obtained from books written by eight authors. The *partial-dataset* was used in this case. The LLNA dynamics was performed until *t* = 500 and the initial states *s*_0_ were defined by a random uniform distribution. The spatio-temporal diagram shows the nodes’ states: dead, in black; and alive, in white. While the horizontal axis represent the nodes (sorted by increasing order of degree *k*, for illustration purposes only), the vertical axis represents the temporal variable.

The dynamic of a network automata provides a global spatio-temporal pattern of evolution. Thus, each network node can be analyzed as a sequence of ones and zeros. Here, we qualitatively discuss the patterns arising from the dynamics of the LLNA modelling 40 books from 8 different authors, which are detailed later in the Dataset section. Fig 3 shows the spatio-temporal diagram of 40 networks using rule B2478-S25. A spatio-temporal diagram is the representation of the states along time, thus, each column represents the state of a given node and each line represents one time step. In this particular case, for each spatio-temporal diagram, the columns were ordered by the node degree. Thus, the leftmost columns are the nodes taking the lowest degrees *k*, and, the right-most, the ones taking the largest values of *k*. Note that the number of nodes *N* varies across networks, therefore, the diagrams are formed by a different number of columns. For simplicity’s sake, the diagrams were scaled to fit within the columns of the table.

Notice that for the particular LLNA rule B2478-S25, Fig 3 reveals a general pattern among all the authors. Three notable regions arise: the leftmost correspond to an oscillatory pattern with a higher tendency of alive nodes, followed by a row with tendency of dead nodes (region comprising nodes with average degree 〈*k*〉 = 3). Then, another shorter oscillatory region appears, followed by a second region, which also presents a higher frequency of dead nodes (region comprising nodes with average degree 〈*k*〉 = 5). The reader should note that rule B2478-S25 does not favor nodes with average degrees 3 and 5 for birth and survival conditions, which explains the distribution of these vertical patterns in the diagrams. The influence of this rule over the nodes with average degree 1 is less apparent due to the lower frequency of these nodes. The rightmost nodes, which correspond to hubs in the network, also show oscillatory patterns that are directly related to the dynamics of rule B2478-S25. This rule favors the birth of nodes and penalizes their survival. Such a behavior illustrates a dependency between rules and network topology.

Despite the above mentioned similar structures in the spatio-temporal diagram, author-dependent patterns can also be noted. For instance, the patterns obtained for Darwin in all five books are strongly similar. Darwin’s textual networks present a bigger region corresponding to nodes with average degree 〈*k*〉 = 3, and a major ratio of nodes with high connectivity which are influenced by the rule. Therefore, the spatio-temporal diagram suggests that the books written by the same author exhibits similar patterns, while allowing to distinguish among the other authors.

#### LLNA measurements

Based on the spatio-temporal diagram, as the ones displayed in Fig 3, several measurements can be used to extract quantitative properties from each individual node that allow to characterize the textual networks in terms of a time series containing only zeros and ones. In this work, we focused on two measurements (the Shannon entropy and Lempel-Ziv complexity) as suggested in the literature [21].

The Shannon entropy of a binary sequence is defined as

$$\begin{array}{c} {\mu_{S}}_{i}=-\left(p_{i}^{0}\log_{2}p_{i}^{0}+p_{i}^{1}\log_{2}p_{i}^{1}\right), \end{array}$$ (9)

where $p_{i}^{1}$ and $p_{i}^{0}$ are the probability of having ones and zeros in the sequence, respectively [53]. The Shannon entropy ranges in the interval [0, 1], where periodic and complex spatio-temporal patterns tend to higher entropy values, while steady patterns tend to lower values.

The Lempel-Ziv complexity *μ*_*L*__*i*_, different from Shannon entropy, is derived from the data compression algorithm proposed by Lempel&Ziv [54]. This measurement is based on the number of different blocks (*g*) that a sequence can contain. A minimum block is defined using the first bit on the left of the sequence. Then, one moves rightward, bit by bit, until an unseen subsequence appears, which is formed starting exactly after a previous block and ending at the current position. For instance, the binary sequence 11110001000111010010 of length *l* = 20, can be divided into *g* = 9 minimum blocks: 1|11|10|0|01|00|011|101|001. Given the number of blocks *g*, the Lempel-Ziv complexity is computed as

$$\begin{array}{c} {\mu_{L}}_{i}=\frac{g\log l}{l}. \end{array}$$ (10)

Similar to the entropy, steady patterns will tend to lower Lempel-Ziv complexities, while more chaotic or randomly patterns will tend to higher values. However, some differences can be found between both measurements. For instances, given two sequences “01010101” and “01001101010111001001”, the first sequence repeats a steady pattern, while the second one seems random. In both cases, the highest entropy is obtained, while their complexities values are 1.30 and 1.35, since they contain 4 and 8 minimum blocks, respectively.

### LLNA-based pattern recognition

We employed the LLNA method to extract the intrinsic patterns from textual networks, which aim to distinguish among authors’ written style. In the so-called training phase, these techniques first identify patterns for each author’s writing style. Then, the patterns identified in the previous phase are used to classify unseen instances in the classification phase. In this manuscript, we setup two systematic frameworks to evaluate the performance of the proposed method: as a one-class and multi-class problem.

In literature, some works have considered the authorship task as a one-class problem [55]. Thus, we addressed the authorship attribution problem by comparing all the collection of books from an author A against the same number of unknown exclusive books from *X* authors. Thus we determine if A is distinguishable from *X*. In this scenario, we used linear classification methods that have been in related text categorization methods, including Naive Bayes (NVB), k Nearest Neighbors (kNN) and Support Vector Machines (SVM), as suggested by Koppel [55].

In addition to the one-class framework, we also evaluated the performance of our method as a multi-class problem. Thus, besides the linear classifiers previously mentioned, several well-known supervised classification methods were also employed: Bayesian Networks (BNT), RBF Networks (RBF), Multi Layer Perceptron (MLP), C4.5 (C45) and Random Forest (RFO) [56]. All classifiers were set up with their default configuration of parameters, as suggested in Ref. [57].

To evaluate the performance of both one-class and multi-class classification schemes, we used the *k*-fold cross-validation strategy [56]. To perform the evaluation, this method splits the data into two sets: the training dataset is the set of samples used for training purposes, while the test set is used for validation purposes. Since these two sets are mutually exclusive and, therefore, the evaluation is performed over unknown instances, the cross-validation method is a reliable strategy. In this study, we use *k* = 5 because each author was characterized by a set of 5 books (see description of the dataset in the next section. Thus, at each iteration, one book of each author is chosen to compound the test dataset, while the remaining books are selected to form the training dataset.

The results were also further probed by using confusion matrices, which are structures, reporting for each possible class (in our case, for each distinct author) the relationship between predicted and real classes. Traditionally, a confusion matrix is used to identify the following patterns of performance: $\alpha_{m_{i},m_{i}}$, which is the number of instances belonging to class *m*_*i*_ which were correctly assigned to *m*_*i*_; while $\alpha_{m_{i},m_{j}}$ is the number of instances belonging to class *m*_*i*_ which were incorrectly assigned to class *m*_*j*_. Specially, the quantity $\alpha_{m_{i},m_{j}}$ will be useful to identify which authors cannot be discriminated with the proposed technique.

### Dataset

An English corpus of known authors (labeled instances in the supervised training phase) was created to evaluate the accuracy of the proposed method. The corpus comprises 100 books in English language, which were obtained from the Project Gutenberg repository [58]. The books in our dataset were written by 20 distinct authors. The full list of books and the respective authors is provided in S4 File of the Supplementary Information. The distribution of books for authors is uniform, i.e. each author is represented by a set of 5 books. In this study, we considered the task of discriminating among 8 distinct authors, namely Doyle, Stoker, Darwin, Dickens, Hardy, Wodehouse, Poe and Munro. This dataset is hereafter referred to as *classification-dataset*. Note that datasets using a similar distribution of authors and genres have been considered in related works [5, 16, 59]. The remaining set of 12 authors, namely Melville, Grey, Lang, Davis, James, Bower, Irving, Wells, Alger, Twain and Hawthorne; hereafter referred to as *rule-selection-dataset*, was used to the particular process of selecting the best LLNA set of rules. The choice of best rules was performed in a different dataset to generate an unbiased classifier [56]. The former dataset was used for rule selection, while the *classification-dataset* was used to evaluate the performance of the classifiers. This procedure ensures that different datasets are employed for the learning and the classification processes. Moreover, due to the *k*-fold cross validation scheme, the training and testing steps were made regarding the *classification-dataset*.

In the general scenario of textual classification, the application of pre-processing steps may be useful for the task in hand. In semantical tasks, such as the word sense disambiguation, the lemmatization of words plays an important role on the performance [60]. In the authorship attribution task, conversely, this same lemmatization step may lead to a great loss of information, hindering the accurate identification of authors’ particular writing choices [4]. However, it has been shown that in network based techniques, the lemmatization step is important to cluster distinct writing forms into the same node. In our experiments, we also evaluated three types of lemmatization strategies to generate the textual networks, which led to the generation of three distinct variations of lemmatization datasets (*none-dataset*, *partial-dataset* and *full-dataset*). The three variations also follow the same methodology detailed before for the creation of both *classification-dataset* and *rule-selection-dataset*. The details of all three variations are summarized below:

1. *none-dataset*: the original dataset was kept, i.e. the lemmatization step was disregarded.
2. *partial-dataset*: the lemmatization was applied only in nouns. Thus, all nouns are mapped to their singular forms.
3. *full-dataset*: the lemmatization was applied to all words. Therefore, verbs and nouns are mapped to their infinitive and singular forms, respectively.

## Results and discussion

The main purpose of this manuscript is to characterize networks representing written texts to obtain informative features for the authorship attribution task. Differently from traditional approaches, here we explored the use of LLNA rules to discriminate network topologies. We have used this approach because it has been shown that authors’ particular writing choices modify word adjacency networks in a consistent form [16].

As described in the Material and methods section, our dataset comprises 100 books written by 20 distinct authors, and three distinct pre-processing strategies were probed to generate the textual networks. Before presenting the results of the classification based on the LLNA approach, we first address the LLNA rule selection, which is detailed in the next section. Thus, we perform the selection of the best LLNA rules using the *rule-selection-dataset* containing the 12 authors. Later, these rules are applied in the authorship problem using the *classification-dataset* comprising the 8 authors. We also compared the proposed approach with the one based on structural measurements [16]. Finally, we explore the effects of the lemmatization process on the properties of the networks.

### LLNA rule selection

The rule selection is as important parameter to achieve higher accuracies using the LLNA method [21]. In fact, the set of Life-like rules can be understood as a parameter that might provide the best classification rates when applied in distinct applications. We evaluated, exhaustively, each of the 262, 144 possible Life-Like rules using the *rule-selection-dataset* comprising 12 authors. As discussed before, the reader should note that the rule selection was performed in different dataset in order to obtain LLNA rules that best represent a true classifier generalization [56].

The LLNA dynamics were evolved during *t* = 400 time steps. To characterize the dynamics of the LLNA, we extracted a feature vector composed by the concatenation of the Shannon entropy and the Lempel-Ziv distributions $\left[{\vec{\mu }}_{S},{\vec{\mu }}_{L}\right]$, which consist of 60 attributes. The first vector contains the distribution of the Shannon entropy *μ*_*S*__*i*_ and the second vector is composed by the Lempel-Ziv complexity distribution ${\vec{\mu }}_{L}$. Both distributions are windowed over 30 bins. Additionally, we performed an analysis of the influence of the two parameters mentioned: the number of time steps and the number of bins of the distributions (see S5 File of the Supplementary Information). We adopted *t* = 400 and the number of bins as 30 in the subsequent experiments performed in this paper, as explained in the next section.

Because the choice of the best rule encompasses the induction and evaluation of 262, 144 classifiers, we only used in this phase the kNN method. We have chosen particularly this method because, in general, it generates better results while keeping an excellent processing time [56]. Note that, the application of other methods in this phase, such as neural networks or SVM, would be impractical owing to the time complexity associated to these methods [56].

**Fig 4:**
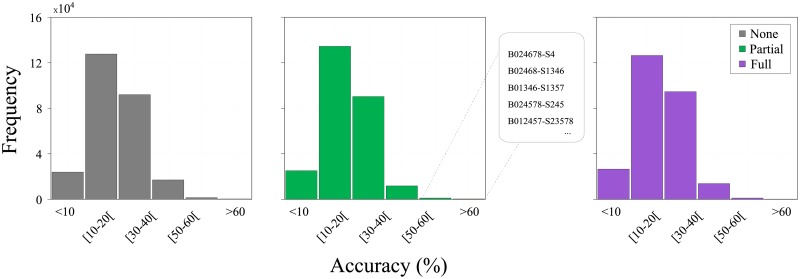
Histogram of the distribution of accuracy. This figure shows the histogram of the distribution of accuracy for all 262, 144 evaluated rules of the LLNA in the *rule-selection-dataset* comprising 12 authors. From left to right, the histograms for each of the 3 datasets *none*, *partial* and *full*, are shown respectively. As an example, the highlighted five rules maximizes the classification of the *rule-selection-dataset*, when a partial lemmatization was applied. For this rule selection experiment, both Shannon entropy and Lempel-Ziv complexity were considered as corresponding feature vectors, and kNN classifier.

Fig 4 depicts the histogram distribution of the accuracies obtained for the complete rule-space of the LLNA. Most of the rules yielded low accuracy classifiers. Typically, accuracies lower than 40% have been found. Conversely, there is a small number of rules that achieved accuracies greater than 60%, corresponding to approximately 50 rules. However, as the rule selection is made through an optimization procedure [21], therefore, in this study, we considered a bigger bunch of solutions, which included also the ones that obtained accuracy rates greater than 55%, which correspond approximately to 400 rules. This strategy is justified since we find better accuracies when tested on the unseen data, as explained later.

Note that the selection of best rules is performed independently in each of three datasets: *none-*, *partial-* and *full-* from the *rule-selection-dataset*. Moreover, as the rule selection is a preliminary phase, one should expect that among the set of best rules further improvement can be achieved by using other LLNA measurements [21].

### Classification of authorship networks

For the authorship identification problem, we applied the best rules obtained to identify authorship in the *classification-dataset* comprising the 8 authors. First, we compared the three datasets, *none-*, *partial-* and *full-dataset* by using different measurements extracted from the LLNA dynamics: the Shannon entropy distribution ${\vec{\mu }}_{S}$ and the Lempel-Ziv distribution ${\vec{\mu }}_{L}$.

We evaluated the performance of the classification by using different LLNA measurements, extracted from the spatio-temporal pattern, in two ways, isolated and combined. Thus, two feature vectors were used to characterize authors’ styles. The first feature vector ${\vec{\mu }}_{S}$ is composed by the distribution of the Shannon entropy *μ*_*S*__*i*_, which is divided into 30 bins, therefore, ${\vec{\mu }}_{S}$ contains 30 attributes. Similarly, the second feature vector ${\vec{\mu }}_{L}$ is composed by the Lempel-Ziv complexity distribution divided into 30 bins. We adopted arbitrarily 30 bins, since this value does not influence the accuracy rates (see S5 File of the Supplementary Information). This vector was normalized by the maximum value achieved among the group of samples. Finally, the combined vector $\left[{\vec{\mu }}_{S},{\vec{\mu }}_{L}\right]$ is the one that concatenates both measurements, which contains 60 attributes.

We tested the accuracy of the 400 selected rules with different feature vectors as well as the combination of them for different classifiers. We emphasize that, as the rule selection is made through an optimization procedure for a specific problem [21], we must assume that the set of solutions (set of selected rules) also bring out some rules that do not fulfill the expectation when presented new dataset. For this reason, we recommended to explore a bigger bunch of solutions (rules), so in this manner we can find better rules.

Table 1 presents the best rules obtained for the *classification-dataset*. The columns ${\vec{\mu }}_{S}$ and ${\vec{\mu }}_{L}$ show the accuracy rates obtained for each distinct feature vector. The results when combining these distributions are shown in the last column of the same table. Note that the isolated feature vector ${\vec{\mu }}_{L}$ yielded the maximum accuracy of 70.5% (± 13.44%) for rule B2478-S25 when using the *partial-dataset*.

**Table 1:** Accuracy rate (%) obtained using different measurements (${\vec{\mu }}_{S}$ and ${\vec{\mu }}_{L}$) and their combinations as attributes ([〈*μ*_*S*_〉, 〈*μ*_*L*_〉]) to classify 8 authors of the *classification-dataset*. To select the best rules, we used the kNN with *k* = 1 and 5-fold cross validation. The best result among all classifiers were also obtained with the kNN method.

Lemmatization	Rule	${\vec{\mu }}_{S}$	${\vec{\mu }}_{L}$	[〈*μ*_*S*_〉, 〈*μ*_*L*_〉]
*None*	B124-S257	51.0(±16.11)	**68.5(±10.90)**	66.0(±12.25)
	B1245-S1245	37.5(±14.88)	62.0(±16.33)	57.5(±16.93)
	B245-S457	61.0(±14.12)	57.5(±17.31)	48.5(±15.44)
*Partial*	B2478-S25	43.5(±14.49)	**70.5(±13.44)**	50.0(±15.31)
	B026-S14	48.5(±15.02)	59.5(±15.00)	63.5(±14.84)
	B148-S6	62.5(±13.01)	36.0(±10.41)	53.5(±15.94)
*Full*	B3567-S03468	39.5(±11.79)	**68.0(±16.57)**	47.5(±20.41)
	B13568-S13	63.5(±15.28)	52.0(±14.74)	34.5(±14.11)
	B0134568-S0123568	36.5(±17.28)	55.0(±14.88)	61.5(±14.40)

**Fig 5:**
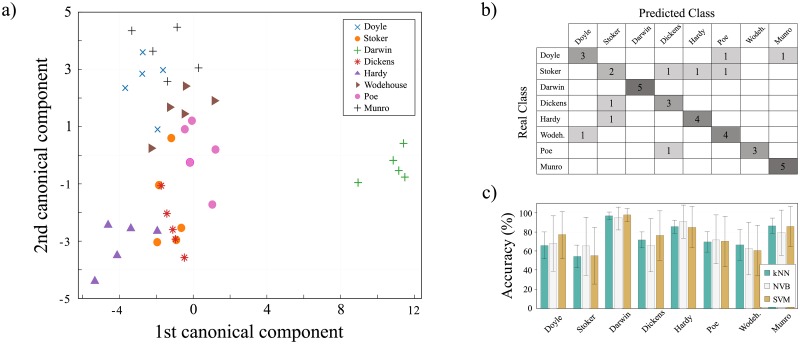
Authorship recognition performance using LLNA. a) Canonical analysis performed for the authorship recognition task using the five books from the authors of the *classification-dataset* using partial lemmatization. For this plot was used rule B2478-S25 and the Lempel-Ziv distribution ${\vec{\mu }}_{L}$ as a feature vector. b) Confusion matrix using kNN method achieved by the best classification rate. Each cell shows the number of correct predicted instances, where nonzero elements are indicated. c) Comparison of the accuracy obtained by the proposed method treating the authorship verification as a one-class classification problem. The accuracy was calculated as the average and standard deviation for the classification of five books of an author A against five books from unknown authors X, using three different classifiers.

To illustrate the discriminability obtained with our method, in Fig 5a, we show a canonical analysis project into two dimensions. In this case, the *partial-dataset* was analyzed, with a dynamics based on the rule B2478-S25 and a characterization performed in terms of the feature vector ${\vec{\mu }}_{L}$. Even though only two dimensions were used to visualize our data, there is a clear separation between Darwin and the other authors. A similar pattern occurs for Hardy, while others can considerably vary their styles from book to book (e.g. Dickens).

In Fig 5b we provide the confusion matrix obtained with the best rule. As expected, Darwin is easily distinguished from the other authors. In a similar fashion, the induced classifier can discriminate among Darwin, Hardy, Wodehouse and Munro. The author with the lowest classification accuracy is Stoker, since three of his books were incorrectly assigned to Dickens, Hardy and Wodehouse.

The best accuracy rate found using the best configuration of parameters shows unequivocally that the proposed features can capture authors’ particularities in written styles, allowing thus the discrimination of authors in unknown texts. Note that a random authorship attribution would accurately recognize authors with probability *p* = 1/8 = 0.125 in our dataset comprising *n*_*b*_ = 40 books. Thus, the *p*-value associated with the obtained accuracy of *n*_*a*_ = 29 books accurately classified (see Fig 5) is

p-value=∑na=29nb(nbna)pna(1-p)(nb-na)≤1.0×10-15, (11)

confirming thus the significance of the obtained results. Furthermore, we also analyzed the authorship task as a one-class problem. Thus, we evaluated the accuracy of an author A against X submitted to a 5-fold cross-validation experiment. As the number of books for all the authors in our validation corpus is five, we randomly choose a book from mutually exclusive authors. In this context there are 21 possible combinations (n=7r=5). Therefore, we determined the accuracies in terms of the average and standard deviation for each author independently. Additionally, we used three different linear classifiers: kNN, NVB and SVM. The results for this experiment are reported in Fig 5c.

### Evaluation of structural measurements and robustness analysis

We compared the results obtained with the Life-Like network automata with structural measurements used to characterize complex networks [16]. We selected the following measurements to compose the feature vector: mean degree (〈*k*〉), average hierarchical degree at the first level ($\langle H_{k_{1}}\rangle$), average hierarchical degree at the second level ($\langle H_{k_{2}}\rangle$), average clustering coefficient (〈*C*〉), average path length (*l*) and degree assortativity (Γ). Each measurement was calculated directly from the textual networks comprising the three datasets (*none*, *partial* and *full*). The left side of Table 2 shows the accuracy obtained in the classification of the network models when using structural measurements. Note that the performance of the structural measurements method, in general, is improved when no lemmatization is applied. The best result was obtained with the SVM classifier (61.30%), which is similar to the best results reported in [16]. A similar performance was also obtained with the MLP classifier (59.23%). The right side of Table 2 shows the results obtained with the proposed method. Rules B124-S257, B2478-S25, B3567-S03468 provided the highest accuracies for the *none-*, *partial-* and *full-dataset* when using only the Lempel-Ziv distribution ${\vec{\mu }}_{L}$.

**Table 2:** Comparison of the accuracy rate (%) obtained using network structural measurements and the proposed method based on network automata. The structural measurements’ feature vector was composed by: mean degree (〈*k*〉), average hierarchical degree of level 1 ($\langle H_{k_{1}}\rangle$), average hierarchical degree of level 2 ($\langle H_{k_{2}}\rangle$), average clustering coefficient (〈*C*〉), average path length (*l*) and degree assortativity (Γ). Remarkably, our method outperforms the latter by an average margin of 9.2%.

	Network structural measurements			Proposed method (LLNA)		
	*None*	*Partial*	*Full*	*None* (B124-S257)	*Partial* (B2478-S25)	*Full* (B3567-S03468)
BNT	48.23(±14.88)	45.43(±14.18)	44.23(±13.62)	41.0(±14.67)	46.0(±13.37)	37.0(±14.20)
NVB	58.28(±15.16)	56.23(±14.5)	51.13(±15.07)	49.0(±12.97)	54.5(±12.95)	48.0(±15.60)
MLP	59.23(±13.92)	50.73(±14.13)	45.03(±15.2)	54.0(±14.31)	59.0(±12.77)	54.0(±12.35)
KNN	52.0(±14.9)	49.40(±14.52)	43.65(±16.38)	68.5(±10.90)	**70.5(±13.44)**	68.0(±16.57)
C45	44.25(±13.05)	42.55(±14.72)	42.13(±14.47)	41.0(±11.13)	45.0(±14.88)	44.5(±13.05)
RFO	53.43(±15.01)	54.60(±14.44)	45.88(±14.18)	57.5(±20.09	51.5(±16.27)	54.5(±12.95)
RBF	52.48(±14.17)	51.68(±14.68)	46.38(±15.52)	46.5(±14.67)	47.0(±12.12)	47.5(±17.68)
SVM	**61.30(±15.56)**	49.28(±13.97)	50.20(±14.70)	43.5(±18.44)	55.5(±13.05)	50.5(±19.59)

**Fig 6:**
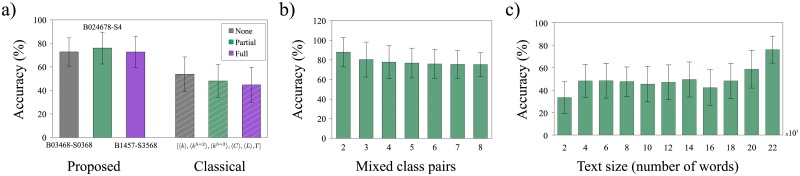
Comparison performance regarding network structural measurements and robustness performance. a) Comparison of the accuracy obtained by the proposed method (left side) and the classical network measurements (right side). The histograms on the left (mean and standard deviation) represent the best accuracies obtained when using rules B124-S257, B2478-S25 and B3567-S03468 for *none-*, *partial-* and *full-dataset*, respectively. In a similar way, the histograms on the right show the best accuracies obtained using the combination of the network measurements: mean degree (〈*k*〉), average hierarchical degree of level 1 ($\langle H_{k_{1}}\rangle$), average hierarchical degree of level 2 ($\langle H_{k_{2}}\rangle$), average clustering coefficient (〈*C*〉), average path length (*l*) and degree assortativity (Γ), as a feature vector. b) Average accuracy obtained in the variations of the original dataset. Each variation considers a different number of authors, which ranges from 2 to 8. c) Performance evaluation for different text size, ranging from 2000 to 22000 words, using rule B2478-S25. For all these experiments kNN method was used.

Considering all datasets and classifiers, the highest accuracy rate was 70.5%. This means that our method outperformed the network structural measurements by a margin of 9.2%, (when compared to the best performance of the structural measurements). The best results obtained by each strategy are also illustrated in Fig 6a. In addition, when comparing the results per datasets, we can see that the performance obtained with LLNA descriptors for k-NN classifier outperforms the structural measurements with a margin of 16.5%, 21.1% and 24.35% for *none*, *partial* and *full* datasets, respectively. For the SVM classifier, only the results for the *none-dataset* were outperformed for the structural measurements.

The robustness of the proposed methodology with regard to the total number of authors was verified by considering other variations of authors in the *classification-dataset*. To do so, we selected all variations of 8 authors among the total of 20 authors. We then applied the proposed methodology to probe the sensibility of our method to specific datasets. As shown in Fig 6b, there is only a minor variation in the accuracy when considering datasets of 8 authors, suggesting that our method is robust with regard to the variation of datasets. A similar procedure was performed to study the robustness in datasets comprising a distinct number of authors (from 2 to 7 authors). Note that, in these other scenarios, a similar robust behavior was found. Interestingly, similar accuracy results have been obtained when considering 3 and 8 authors, suggesting thus that our method is more effective when more complex authorship attribution tasks are considered.

Furthermore, as in real world authorship attribution problems, the size of a text is an issue, we also evaluated the tolerance of our proposed method with regard to the number of words within the books. We explored different bunch of words (2000, 4000 …, 22000). The accuracies obtained using the kNN classifier are shown in Fig 6c. According to the results, we observed that the performance is hampered for shorten texts, however for reasonable text size the accuracy is improved.

### Effect of the lemmatization on network measurements

Table 3 shows the topological properties for one of Doyle’s book modeled as a network, considering the three lemmatization processes (*none*, *partial* and *full*). See the complete set of measurements in S6 File of the Supplementary Information. The columns show the measurements presented in the Material and methods section, as follows: number of nodes *N*, number of edges *E*, average degree 〈*k*〉, clustering coefficient 〈*C*〉, average path length 〈*L*〉, power-law exponent *γ*, diameter *D*, density *d* and degree assortativity Γ.

**Table 3:** Measurements extracted for the textual network corresponding to Doyle’s book “*Uncle Bernac—A Memory of the Empire*” regarding the three types of lemmatization process (*none-*, *partial-* and *full-dataset*).

Lemm.	*N*	*E*	〈*k*〉	〈*C*〉	〈*L*〉	*γ*	*D*	*d*	Γ
*None*	5914	22991	7.78	0.04	3.63	2.33	11	0.0013	-0.06
*Partial*	5374	22775	8.48	0.04	3.54	2,29	11	0.0016	-0.06
*Full*	4977	22451	9.02	0.05	3.47	2.20	10	0.0018	-0.07

From the same table, one can note a decreasing of both the number of nodes *N* and edges *E*, while the average degree 〈*k*〉 increases. This effect can be explained by the fact that when the lemmatization process is performed, the multiple representations of a word are all transformed to its canonical form, e.g., the words *has* and *have* will have only one representation in a network, the node *have*, instead of having two. Moreover, the diameter for all the networks is maintained around 11. We also observed that all networks studied here obey a power law constant around ≈ 2.27. Therefore, these textual networks have a scale-free structure, which is supported by the maximum likelihood method and the Kolmogorov-Smirnov statistic that accepts the hypotheses of a reasonable fit. Moreover, this property is consistent with the scale-free textual networks found in the literature.

**Fig 7:**
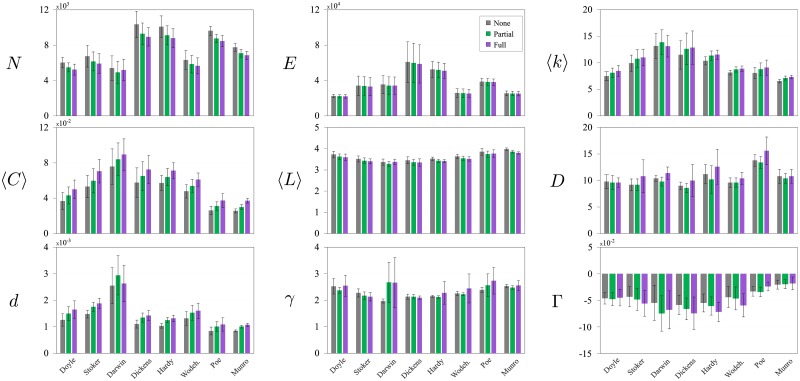
Average network measurements. Network structural measurement extracted from eight authors highlighted in the diagrams and for the three datasets: *none-*, *partial-* and *full-dataset* (see description in the Material and methods section). The following distributions are shown for each author: number of nodes (*N*), number of edges (*E*), average connectivity (〈*k*〉), average clustering coefficient (〈*C*〉), average path length (〈*L*〉), diameter (*D*), density (*d*), power-law exponent (*γ*) and degree assortativity (Γ).

Fig 7 presents a set of average topological measurements calculated for each author of the *classification-dataset*. The standard deviation was obtained considering the five books of each author. Fig 7 also shows the values obtained for the three variations of dataset. The main results concerning each measurement are described below:

- **Total number of nodes (*N*) and edges (*E*)**: *N* decreases with the lemmatization process, whereas *E* is not influenced by this process. This effect occurs because, even when nodes are removed during the lemmatization, adjacency relationships are not affected, and, consequently, the degree of the remaining nodes tends to increase. This effect is evident in the top-right diagram displaying the average network connectivity 〈*k*〉.
- **Average clustering coefficient (〈*C*〉)**: This measurement was influenced by both *N* and *k*. 〈*C*〉 tends to increase with the lemmatization process because the network remains with almost the same number of edges, while the number of nodes decreases as a consequence of mapping distinct variations of the same concept into the same node.
- **Average shortest path length (〈*L*〉)**: Similarly to the number of edges, the average shortest path length is not much affected by the lemmatization process. However, note that the values of 〈*L*〉 tend to decrease as a consequence of the decrease in the total number of nodes.
- **Diameter (*D*)**: In most cases, the diameter increases by a short margin when the lemmatization process is performed. However, this pattern seems to depend from author to author. Note, e.g. that the average diameter decreases when the full lemmatization is applied for books authored by Doyle. Conversely, the lemmatization process seems to cause an opposite effect on networks modelling books written by Allan Poe.
- **Density (*d*)**: The density of links increases in most cases, as the lemmatization process removes nodes, and the number of edges is practically not affected. An exception occurs for Darwin. Remarkably, the average density of the *none-* and *full-* datasets are in a similar fashion.
- **Power-law exponent (*γ*)**: Almost all the textual networks present power exponent between 2 and 3, which is a characteristic that have been demonstrated for many real-world networks [45, 61] and, particularly in text networks, is a consequence of the Zipf’s Law. Concerning the effect of the lemmatization process on this feature, no clear pattern can be identified, as opposite effects have been found e.g. for Stoker and Poe.

## Conclusion

In this paper, we have addressed the authorship attribution problem, which is a task of practical relevance in many contexts of information science research. We have specifically studied the effect of the textual organization in the discriminability of documents written by distinct authors. To capture the structural properties of texts, we have used the well-known network framework, given its potential revealed in related applications. Unlike the approaches based only on topological properties of networks, we have proposed here a methodology to capture further information concerning authors’ particular styles. To do so, we have represented networks modelling texts as network automata with a dynamics based on Life-Like rules. The LLNA method searches the whole rule space for an optimal solution to one specific problem. The best rule for a single dataset may not perform as well as for a second dataset. Therefore, there is no generalization regarding the optimization procedure of finding the best Life-Like rule. This is not a limitation of the method, however this issue reflects intrinsic characteristics of each data source. Consequently, the selection of the best rules has to be performed per dataset. The results presented in this paper are supported by the rule optimization procedure which was performed for the dataset of interest aiming at solving a specific authorship attribution problem. The insertion of new authors in this dataset would require a new training procedure. Upon selecting a set of discriminative rules that serve to coordinate the automata dynamics, we have found that the variations in the binary states of nodes are more discriminative than simple network structural measurements approach. More specifically, we have outperformed the latter approach in 9.2% for the classification of 8 distinct authors. Interestingly, the best results were obtained with a partial lemmatization process, suggesting that this procedure is more adequate than just lemmatizing all words when text networks are used as the underlying model for this task.

The methodology proposed here paves the way for improving the characterization of related information systems modelled in terms of networks. This is evident if we recall that network automata approaches are specially suitable to describe networks with scale-free distributions [21] and, as a consequence, documents following Zipf’s Law. Further works could investigate the effectiveness of our approach e.g. in the analysis of the complexity of texts or in applications related to extractive summarization. Given the complementarity of the analysis provided by the network automata framework, we argue that the combination of the proposed technique with those relying on traditional superficial features [62–65] could lead to optimized results, since word adjacency networks are oftentimes used as an additional tool in natural language processing problems.

## Supporting information

S1 File List of stopwords and preprocessing steps. (PDF)

S2 File Example of network construction. (PDF)

S3 File Illustration of LLNA rule B3/S23. (PDF)

S4 File List of books, authors and networks datasets. (ZIP)

S5 File Analysis and selection of parameters time *t* and number of bins. a) Accuracy (%) in relation to the evolved time *t*. b) Accuracy (%) for different number of bins to compose the feature vector using the Lempel-Ziv complexity distributions (*μ*_*L*_). Both experiments in a) and b) were made using rule B2478-S25 and the *partial-dataset* and classifier kNN with k = 1 and 5-fold cross validation.
(PDF)

S6 File Network measurements for the three datasets. (XLSX)
