# Supplementary material for: Authorship attribution based on Life-Like Network Automata
Source: PLoS One. 2018 Mar 22;13(3):e0193703. doi: 10.1371/journal.pone.0193703 (PMC5863954; doi:10.1371/journal.pone.0193703)
Supplement: S1 File — (PDF) [file pone.0193703.s001.pdf]

Authorship attribution based on Life-Like network automata - Supplementary Information

Jeaneth Machicao[1+], Edilson A. Correa Jr.[2] , Gisele H. B. Miranda[2] , Diego R. Amancio[2], and Odemir M. Bruno[1,2,+]

1 Sao Carlos Institute of Physics, University of São Paulo, São Carlos - SP, PO Box 369, 13560-970, Brazil.
2 Institute of Mathematics and Computer Science, University of Sao Paulo, São Carlos - SP, 13560-970, Brazil.
* Corresponding author: bruno@ifsc.usp.br

S1 File. List of stopwords, preprocessing steps

1. List of stopwords

The follwing list of stopwords was used in our experiments:

’i’, ’me’, ’my’, ’myself’, ’we’, ’our’, ’ours’, ’ourselves’, ’you’, ’your’, ’yours’, ’yourself’, ’yourselves’, ’he’, ’him’, ’his’, ’himself’, ’she’, ’her’, ’hers’, ’herself’, ’it’, ’its’, ’itself’, ’they’, ’them’, ’their’, ’theirs’, ’themselves’, ’what’, ’which’, ’who’, ’whom’, ’this’, ’that’, ’these’, ’those’, ’am’, ’is’, ’are’, ’was’, ’were’, ’be’, ’been’, ’being’, ’have’, ’has’, ’had’, ’having’, ’do’, ’does’, ’did’, ’doing’, ’a’, ’an’, ’the’, ’and’, ’but’, ’if’, ’or’, ’because’, ’as’, ’until’, ’while’, ’of’, ’at’, ’by’, ’for’, ’with’, ’about’, ’against’, ’between’, ’into’, ’through’, ’during’, ’before’, ’after’, ’above’, ’below’, ’to’, ’from’, ’up’, ’down’, ’in’, ’out’, ’on’, ’off’, ’over’, ’under’, ’again’, ’further’, ’then’, ’once’, ’here’, ’there’, ’when’, ’where’, ’why’, ’how’, ’all’, ’any’, ’both’, ’each’, ’few’, ’more’, ’most’, ’other’, ’some’, ’such’, ’no’, ’nor’, ’not’, ’only’, ’own’, ’same’, ’so’, ’than’, ’too’, ’very’, ’s’, ’t’, ’can’, ’will’, ’just’, ’don’, ’should’, ’now’

2. Pre-processing steps

To illustrate each step of the pre-processing phase, consider the following text:

Twice already in his career had Holmes helped him to attain success, his own sole reward being the intellectual joy of the problem. For this reason, the affection and respect of the Scotchman for his amateur colleague were profound, and he showed them by the frankness with which he consulted Holmes in every difficulty. Mediocrity knows nothing higher than itself; but talent instantly recognizes genius, and MacDonald had talent enough for his profession to enable him to perceive that there was no humiliation in seeking the assistance of one who already stood alone in Europe, both in his gifts and in his experience. Holmes was not prone to friendship, but he was tolerant of the big Scotchman, and smiled at the sight of him.

The first step is the ***tokenization process***. This step accounts for splitting the text into meaningful segments:

’Twice’, ’already’, ’in’, ’his’, ’career’, ’had’, ’Holmes’, ’helped’, ’him’, ’to’, ’attain’, ’success’, ’his’, ’own’, ’sole’, ’reward’, ’being’, ’the’, ’intellectual’, ’joy’, ’of’, ’the’, ’problem’, ’For’, ’this’, ’reason’, ’the’, ’affection’, ’and’, ’respect’, ’of’, ’the’, ’Scotchman’, ’for’, ’his’, ’amateur’, ’colleague’, ’were’, ’profound’, ’and’, ’he’, ’showed’, ’them’, ’by’, ’the’, ’frankness’, ’with’, ’which’, ’he’, ’consulted’, ’Holmes’, ’in’, ’every’, ’difficulty’, ’Mediocrity’, ’knows’, ’nothing’, ’higher’, ’than’, ’itself’, ’but’, ’talent’, ’instantly’, ’recognizes’, ’genius’, ’and’, ’MacDonald’, ’had’, ’talent’, ’enough’, ’for’, ’his’, ’profession’, ’to’, ’enable’, ’him’, ’to’, ’perceive’, ’that’, ’there’, ’was’, ’no’, ’humiliation’, ’in’, ’seeking’, ’the’, ’assistance’, ’of’, ’one’, ’who’, ’already’, ’stood’, ’alone’, ’in’, ’Europe’, ’both’, ’in’, ’his’, ’gifts’, ’and’, ’in’, ’his’, ’experience’, ’Holmes’, ’was’, ’not’, ’prone’, ’to’, ’friendship’, ’but’, ’he’, ’was’, ’tolerant’, ’of’, ’the’, ’big’, ’Scotchman’, ’and’, ’smiled’, ’at’, ’the’, ’sight’, ’of’, ’him’.

The ***removal of stopwords*** is performed to create a network devoid of words conveying little semantic meaning:

’twice’, ’already’, ’career’, ’holmes’, ’helped’, ’attain’, ’success’, ’sole’, ’reward’, ’intellectual’, ’joy’, ’problem’, ’reason’, ’affection’, ’respect’, ’scotchman’, ’amateur’, ’colleague’, ’profound’, ’showed’, ’frankness’, ’consulted’, ’holmes’, ’every’, ’difficulty’, ’mediocrity’, ’knows’, ’nothing’, ’higher’, ’talent’, ’instantly’, ’recognizes’, ’genius’, ’macdonald’, ’talent’, ’enough’, ’profession’, ’enable’, ’perceive’, ’humiliation’, ’seeking’, ’assistance’, ’one’, ’already’, ’stood’, ’alone’, ’europe’, ’gifts’, ’experience’, ’holmes’, ’prone’, ’friendship’, ’tolerant’, ’big’, ’scotchman’, ’smiled’, ’sight’ Finally, the remaining words are lemmatized, i.e. selected words are transformed into their canonical form (e.g.: absorbed 7→ absorb). Note that this procedure is not applied to the none-dataset. ’twice’, ’already’, ’career’, ’holmes’, ’help’, ’attain’, ’success’, ’sole’, ’reward’, ’intellectual’, ’joy’, ’problem’, ’reason’, ’affection’, ’respect’, ’scotchman’, ’amateur’, ’colleague’, ’profound’, ’show’, ’frankness’, ’consult’, ’holmes’, ’every’, ’difficulty’, ’mediocrity’, ’know’, ’nothing’, ’high’, ’talent’, ’instantly’, ’recognize’, ’genius’, ’macdonald’, ’talent’, ’enough’, ’profession’, ’enable’, ’perceive’, ’humiliation’, ’seek’, ’assistance’, ’one’, ’already’, ’stand’, ’alone’, ’europe’, ’gift’, ’experience’, ’holmes’, ’prone’, ’friendship’, ’tolerant’, ’big’, ’scotchman’, ’smile’, ’sight’
