# Supplementary material for: Authorship attribution based on Life-Like Network Automata
Source: PLoS One. 2018 Mar 22;13(3):e0193703. doi: 10.1371/journal.pone.0193703 (PMC5863954; doi:10.1371/journal.pone.0193703)
Supplement: S2 File — (PDF) [file pone.0193703.s002.pdf]

Authorship attribution based on Life-Like network automata - Supplementary Information

Jeaneth Machicao[1+], Edilson A. Correa Jr.[2] , Gisele H. B. Miranda[2] , Diego R. Amancio[2], and Odemir M. Bruno[1,2,+]

1 Sao Carlos Institute of Physics, University of São Paulo, São Carlos - SP, PO Box 369, 13560-970, Brazil.
2 Institute of Mathematics and Computer Science, University of Sao Paulo, São Carlos - SP, 13560-970, Brazil.
* Corresponding author: bruno@ifsc.usp.br

S2 File. Network construction

An exemplification of the network construction from an already pre-processed text extracted from the book The Valley of Fear by Doyle is shown in the figure below

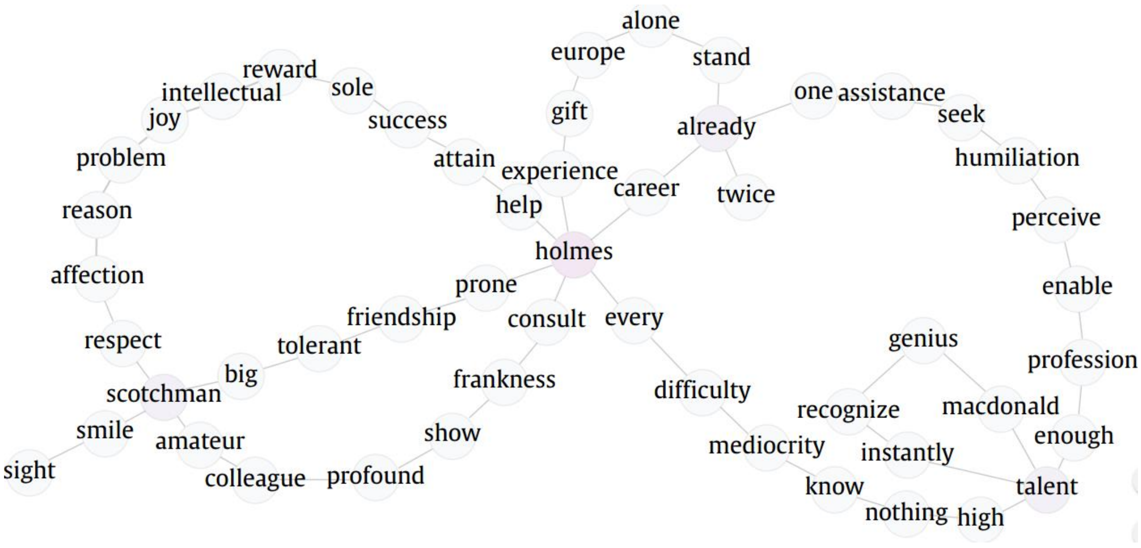

Exemplification of the network modeling using a short text extracted from ''The Valley of Fear'' by Arthur Conan Doyle. In this example, we considered the lemmatization of all words to construct the network.
