# Supplementary material for: Authorship attribution based on Life-Like Network Automata
Source: PLoS One. 2018 Mar 22;13(3):e0193703. doi: 10.1371/journal.pone.0193703 (PMC5863954; doi:10.1371/journal.pone.0193703)
Supplement: S3 File — (PDF) [file pone.0193703.s003.pdf]

Authorship attribution based on Life-Like network automata - Supplementary Information

Jeaneth Machicao[1+], Edilson A. Correa Jr.[2] , Gisele H. B. Miranda[2] , Diego R. Amancio[2], and Odemir M. Bruno[1,2,+]

1 Sao Carlos Institute of Physics, University of São Paulo, São Carlos - SP, PO Box 369, 13560-970, Brazil.
2 Institute of Mathematics and Computer Science, University of Sao Paulo, São Carlos - SP, 13560-970, Brazil.
* Corresponding author: bruno@ifsc.usp.br

S3 File. Illustration of LLNA rule B3/S23

$$s(c_i, t+1) = \begin{cases} 1, & \text{if } s(c_i,t) = 0 \text{ and } 3/r \leq \rho_i < 4/r \\ 1, & \text{if } s(c_i,t) = 1 \text{ and } (2/r \leq \rho_i < 3/r \text{ or } 3/r \leq \rho_i < 4/r) \\ 0, & \text{otherwise.} \end{cases}$$

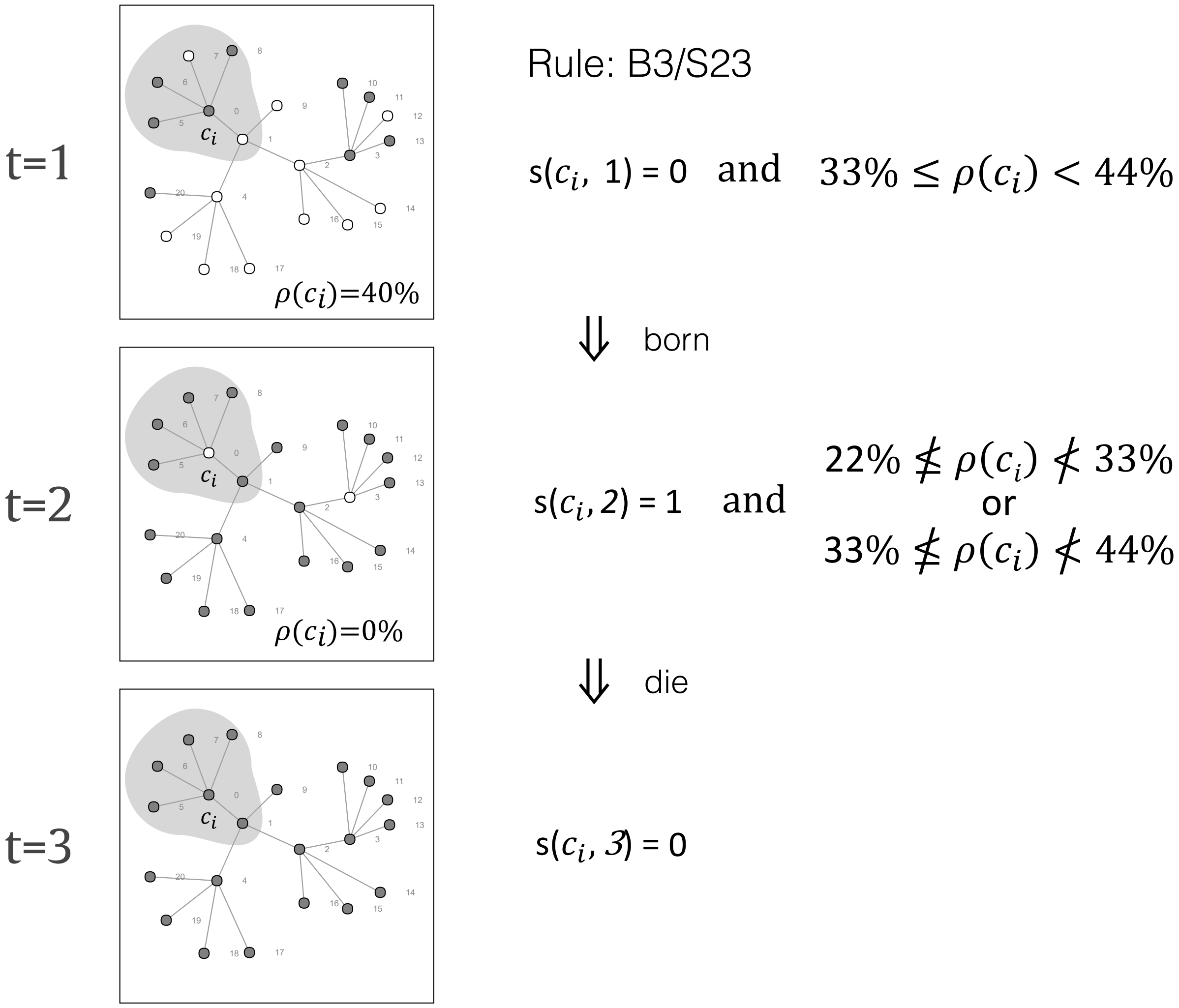

For more information see http://scg.ifsc.usp.br/llna/
