# Supplementary material for: Authorship attribution based on Life-Like Network Automata
Source: PLoS One. 2018 Mar 22;13(3):e0193703. doi: 10.1371/journal.pone.0193703 (PMC5863954; doi:10.1371/journal.pone.0193703)
Supplement: S5 File — a) Accuracy (%) in relation to the evolved time t. b) Accuracy (%) for different number of bins to compose the feature vector using the Lempel-Ziv complexity distributions (μL). Both experiments in a) and b) were made using rule B2478-S25 and the partial-dataset and classifier kNN with k = 1 and 5-fold cross validation. (PDF) [file pone.0193703.s005.pdf]

Authorship attribution based on Life-Like network automata - Supplementary Information

Jeaneth Machicao[1+], Edilson A. Correa Jr.[2], Gisele H. B. Miranda[2], Diego R. Amancio[2], and Odemir M. Bruno[1,2,+]

1 Sao Carlos Institute of Physics, University of São Paulo, São Carlos - SP, PO Box 369, 13560-970, Brazil.
2 Institute of Mathematics and Computer Science, University of Sao Paulo, São Carlos - SP, 13560-970, Brazil.
* Corresponding author: bruno@ifsc.usp.br

S5 File. Analysis and selection of parameters time t and number of bins

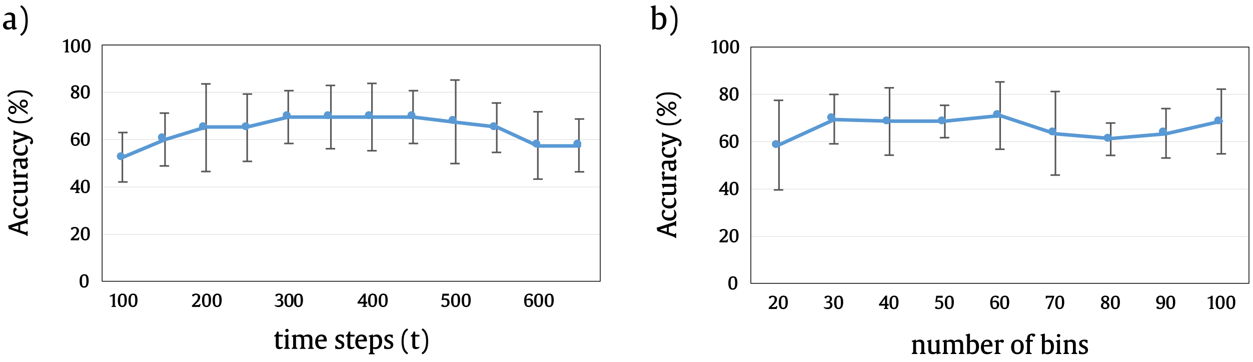

a) Accuracy (%) in relation to the evolved time t. b) Accuracy (%) for different number of bins to compose the feature vector using the Lempel-Ziv complexity distributions (μ_L). Both experiments in a) and b) were made using rule B2478-S25 and the *partial-dataset* and classifier kNN with k=1 and 5-fold cross validation.
