# Supplementary material for: Authorship attribution based on Life-Like Network Automata
Source: PLoS One. 2018 Mar 22;13(3):e0193703. doi: 10.1371/journal.pone.0193703 (PMC5863954; doi:10.1371/journal.pone.0193703)
Supplement: S4 File — (ZIP) [file pone.0193703.s004.zip › S4_File/S4_File.zip!/S4_File/S4_File.pdf]

# Authorship attribution based on Life-Like network automata - Supplementary Information

Jeaneth Machicao[1+], Edilson A. Correa Jr.[2] , Gisele H. B. Miranda[2] , Diego R. Amancio[2], and Odemir M. Bruno[1,2,+]

1 Sao Carlos Institute of Physics, University of São Paulo, São Carlos - SP, PO Box 369, 13560-970, Brazil.
2 Institute of Mathematics and Computer Science, University of Sao Paulo, São Carlos - SP, 13560-970, Brazil.
* Corresponding author: bruno@ifsc.usp.br

## S4 File. List of books and authors

## Validation-dataset

Author 1 - Arthur Conan Doyle (1859-1930)

- Book 1: Uncle Bernac - A Memory of the Empire
- Book 2: The Tragedy of the Korosko
- Book 3: The Valley of Fear
- Book 4: The War in South Africa
- Book 5: Through the Magic Door

Author 2 - Bram Stoker (1847-1912)

- Book 1: Dracula's Guest
- Book 2: The Jewel Of Seven Stars
- Book 3: The Lady of the Shroud
- Book 4: Lair of the White Worm
- Book 5: The Man

Author 3 - Charles Darwin (1809-1882)

- Book 1: Coral Reefs
- Book 2: The Expression of the Emotions in Man and Animals
- Book 3: Geological Observations on South America
- Book 4: The Different Forms of Flowers on Plants of the Same Species
- Book 5: Volcanic Islands

Author 4 - Charles Dickens (1812-1870)

- Book 1: American Notes
- Book 2: A Tale of Two Cities
- Book 3: Hard Times
- Book 4: Great Expectations
- Book 5: Barnaby Rudge: A Tale of the Riots of Eighty

Author 5 - Thomas Hardy (1840-1928)

- Book 1: A Changed Man; and Other Tales
- Book 2: A Pair of Blue Eyes
- Book 3: Far from the Madding Crowd
- Book 4: Jude the Obscure
- Book 5: The Hand of Ethelberta

Author 6 - Pelham Grenville Wodehouse (1881-1975)

- Book 1: My Man Jeeves
- Book 2: Tales of St. Austin’s
- Book 3: The Adventures of Sally
- Book 4: The Clicking of Cuthbert
- Book 5: The Man with Two Left Feet

Author 7 - Alan Poe (1809-1849)

- Book 1: The Works of Edgar Allan Poe (Volume 1)
- Book 2: The Works of Edgar Allan Poe (Volume 2)
- Book 3: The Works of Edgar Allan Poe (Volume 3)
- Book 4: The Works of Edgar Allan Poe (Volume 4)
- Book 5: The Works of Edgar Allan Poe (Volume 5)

Author 8 - Hector Hugh Munro (Saki) (1870-1916)

- Book 1: Beasts and Super Beasts
- Book 2: The Chronicles of Clovis
- Book 3: The Toys of Peace
- Book 4: When William Came
- Book 5: The Unbearable Bassington

## Rule-selection-dataset

Author 9 - Herman Melville (1819-1891)

- Book 1: Moby Dick; Or, The Whale
- Book 2: The Piazza Tales
- Book 3: The Confidence-Man: His Masquerade
- Book 4: Typee: A Romance of the South Seas
- Book 5: White Jacket; Or, The World on a Man-of-War

Author 10 - Zane Grey (1872-1939)

- Book 1: The Lone Star Ranger: A Romance of the Border
- Book 2: Riders of the Purple Sage
- Book 3: The Mysterious Rider
- Book 4: To the Last Man
- Book 5: The Call of the Canyon

Author 11 - Andrew Lang (1844-1912)

- Book 1: The Blue Fairy Book
- Book 2: The Arabian Nights Entertainments
- Book 3: The Yellow Fairy Book
- Book 4: The Violet Fairy Book
- Book 5: The Pink Fairy Book

Author 12 - Richard Harding Davis (1864-1916)

- Book 1: Real Soldiers of Fortune
- Book 2: Notes of a War Correspondent
- Book 3: Cinderella, and Other Stories
- Book 4: The Congo and Coasts of Africa
- Book 5: Soldiers of Fortune

Author 13 - Henry James (1843-1916)

- Book 1: The Turn of the Screw
- Book 2: The Portrait of a Lady - Volume 1
- Book 3: The Ambassadors

- Book 4: The Real Thing and Other Tales
- Book 5: The American

Author 14 - B. M. Bower (1871-1940)

- Book 1: Cabin Fever
- Book 2: The Trail of the White Mule
- Book 3: Lonesome Land
- Book 4: The Lookout Man
- Book 5: The Long Shadow

Author 15 - Washington Irving (1783-1859)

- Book 1: The Sketch-Book of Geoffrey Crayon
- Book 2: Knickerbocker's History of New York, Complete
- Book 3: Chronicle of the Conquest of Granada, from the mss. of Fray Antonio Agapida
- Book 4: The Alhambra
- Book 5: Tales of a Traveller

Author 16 - Herbert George Wells (1866-1946)

- Book 1: The War of the Worlds
- Book 2: A Short History of the World
- Book 3: The First Men in the Moon
- Book 4: Tales of Space and Time
- Book 5: The World Set Free

Author 17 - Horatio Alger (1832-1899)

- Book 1: Ragged Dick, Or, Street Life in New York with the Boot-Blacks
- Book 2: Adrift in New York: Tom and Florence Braving the World
- Book 3: Brave and Bold; Or, The Fortunes of Robert Rushton
- Book 4: Fame and Fortune; or, The Progress of Richard Hunter
- Book 5: The Errand Boy; Or, How Phil Brent Won Success

Author 18 - Jane Austen (1775-1817)

- Book 1: Pride and Prejudice
- Book 2: Emma
- Book 3: Sense and Sensibility
- Book 4: Persuasion
- Book 5: Mansfield Park

Author 19 - Mark Twain (1835-1910)

- Book 1: Adventures of Huckleberry Finn
- Book 2: The Adventures of Tom Sawyer
- Book 3: The Prince and the Pauper
- Book 4: A Connecticut Yankee in King Arthur's Court
- Book 5: Roughing It

Author 20 - Nathaniel Hawthorne (1804-1864)

- Book 1: The Scarlet Letter
- Book 2: Mosses from an Old Manse, and Other Stories
- Book 3: The House of the Seven Gables
- Book 4: Twice Told Tales
- Book 5: The Blithedale Romance
